# The HSN egg-laying command neurons regulate the defecation motor program in *Caenorhabditis elegans*: Integration

**DOI:** 10.17912/micropub.biology.000095

**Published:** 2019-03-29

**Authors:** Bhavya Ravi, Jessica Garcia, Kevin M Collins

**Affiliations:** 1 Neuroscience Program, Miller School of Medicine, University of Miami, Miami, FL 33136; 2 Department of Biology, University of Miami, Coral Gables, FL 33146; 3 Present address: Department of Neurology and Neuroscience, Johns Hopkins University School of Medicine, Baltimore, MD 21205

**Figure 1:**
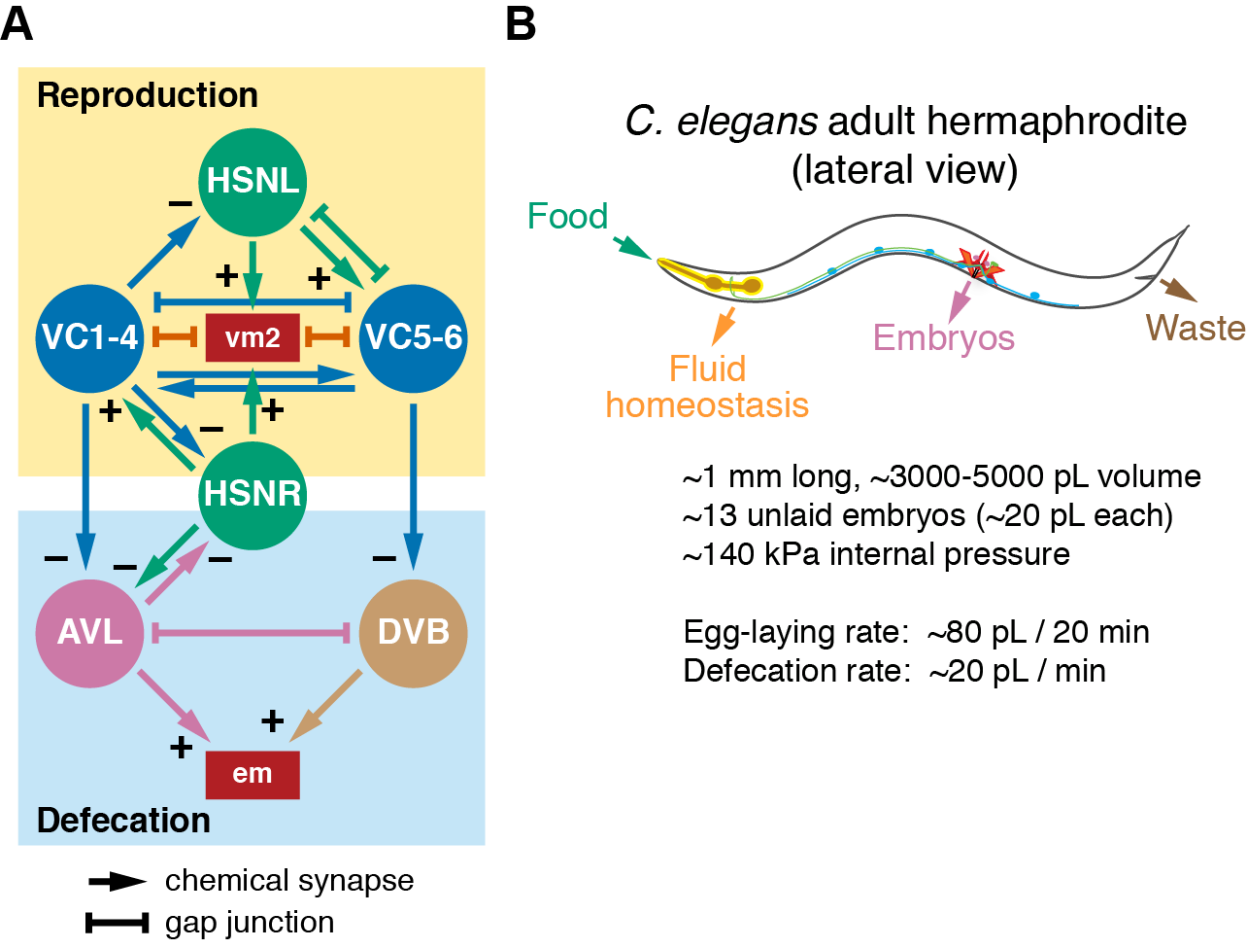
(**A**) Wiring diagrams of the reproductive circuit (top) and defecation motor circuit (bottom). HSN (green) and VC (blue) neurons synapse onto each other and the vm2 muscles for egg laying. Data from White J.G. et al. (1986) indicate HSN and VC also make and receive synapses from AVL and DVB, excitatory GABA motor neurons that regulate the contraction of the enteric muscles (em) for defecation. Arrows indicate chemical synapses, and + or – indicates a presumptive excitatory or inhibitory synapse, respectively. Bar-headed lines indicate gap junctions (e.g. electrical synapses). **(B)** Model summarizing a mass and pressure balance for *C. elegans*. Bacterial food is consumed through the pharynx and used for worm growth. Waste is expelled via defecation through the anus, while animal reproduction occurs through embryo release through the vulva. Fluid homeostasis is maintained via the excretory pore. Also listed are measurements of size (Bolanowski *et al.* 1981), internal pressure (Gilpin *et al.* 2015), and estimated volumes of expulsion events and their frequency.

## Description

We have identified a relationship between egg-laying and defecation behaviors in *C. elegans*. As shown in Figure 1A, the egg-laying and defecation motor circuits show synaptic connectivity. The HSN command neurons and VC motor neurons make and receive synapses from the excitatory GABAergic AVL and DVB motoneurons that regulate defecation (White, J.G. *et al.* 1986). Serotonin and Ga_o_ signaling, which regulate egg laying behavior, can also signal to inhibit defecation (Ségalat *et al.* 1995; Waggoner *et al.* 1998; Tanis *et al.* 2008). Because evidence shows that both the egg-laying active state and the defecation motor program (DMP) are both linked to changes in forward and reverse locomotion (Hardaker *et al.* 2001; Nagy *et al.* 2015), we reasoned there may be a similar relationship between expulsive behaviors that drive either egg laying or defecation. Our experiments document an association between HSN Ca^2+^ activity and a reduced frequency of defecation (Ravi and Collins 2019) *[See accompanying microPub Ravi and Collins (I) 2019]*. Animals lacking HSNs have a reduced defecation frequency (Garcia and Collins 2019) *[See accompanying microPub Garcia and Collins (II) 2019]*. We hypothesize that egg-laying and defecation behaviors are coordinated because they use the same internal hydrostatic pressure to drive expulsion of uterine or intestinal contents, respectively.

Worms continuously internalize bacterial food via pumping of a muscular pharynx (Figure 1B; Avery and Horvitz, 1989). Despite this continuous intake of mass, worms maintain a relatively uniform size, shape, and an internal hydrostatic pressure of ~140 kPa (Knight *et al.* 2002; Gilpin *et al.* 2015; Fechner *et al.* 2018), releasing waste about once per minute and ~3-5 fertilized eggs (~20 pL each) about every 20 minutes (Liu and Thomas 1994; Waggoner *et al.* 1998). During defecation, sequential activity of the anterior and posterior body wall muscles contracts the animal, increasing internal pressure that drives expulsion of liquid waste through the anus (Thomas 1990; Reiner *et al.* 1995). Mutations that eliminate the defecation motor program still expel gut contents at much reduced frequency. This is thought to be caused by a gradual accumulation of internal pressure by ongoing pharyngeal pumping of food that eventually ejects waste through the anus independent of circuit activity or muscle contractility (Avery and Thomas 1997).

Our recent data suggest egg-laying behavior is regulated by a stretch-dependent homeostat. Feedback from embryo accumulation in the uterus activates the postsynaptic muscles which drives burst-firing in the presynaptic HSNs as visualized by Ca^2+^ imaging in behaving animals (Ravi *et al.* 2018a; b). Animals lacking HSNs still enter active states with strong vulval muscle contractions driving release of embryos which additionally supports this model (Collins *et al.* 2016). Electrical silencing of the postsynaptic muscles renders animals egg-laying defective with embryos often hatching inside the mother (Reiner *et al.* 1995). Unlike gut contents which are more fluid, fertilized embryos are more mechanically rigid, requiring full opening of the vulva for efficient release (Li *et al.* 2013). We propose that changes in the internal hydrostatic pressure that accompany food consumption and embryo production activate mechanoreceptors that facilitate the onset of defecation and egg-laying behaviors. As animals age, they continue to eat and grow larger, but their defecation frequency decreases (Bolanowski *et al.* 1981). Egg laying frequency also increases with age for as long as animals have sufficient sperm for oocyte fertilization (McCarter *et al.* 1999). This increase in egg laying in older adults reflects both an increase in the number of eggs expelled with each vulval opening and longer active behavior states. We propose that the timing of expulsive behaviors including defecation and egg laying is regulated by sensory mechanisms that detect changes in internal pressure and/or stretch to maintain homeostasis. Feedback of successful egg laying might also signal to the germ line to ensure the continued production of oocytes for fertilization.
